# MPdb: Melghat Plant databank

**DOI:** 10.6026/973206300181119

**Published:** 2022-12-31

**Authors:** Nitin M Atre1, Purna N Shrimali, Dinesh D Khedkar

**Affiliations:** 1 Bioinformatics and Data Management, ICMR - National Institute of Virology, Pune, Maharashtra, India; 2 Department of Computer Science, Shri Shivaji Science College, Amravati, Maharashtra, India; 3 Department of Botany, Shri Shivaji Science College, Amravati, Maharashtra, India

**Keywords:** MPdb, Melghat plant database, Melghat flora, medicinal plants, Melghat

## Abstract

Melghat Plant databank (MPdb) is the first attempt to review all the past floristic documents and medicinal plants and digitize the Melghat Flora. This curated database contains compiled information about 1028 plants from 139 plant families and 537 genera. Curated medicinal plant information gathered 660 medicinal species records from 128 plant families and 421 genera in MPdb for Melghat Flora. Each plant record is reviewed, scrutinizes, and recorded. More than 9000 records for medicinal uses for nearly 140 diseases, reported phytochemicals, and published cross references. MPdb will serve as a valuable information resource for students, researchers, and aboriginal communities to explore Melghat Flora for better-applied prospects in herbal drug research.

## Background:

In the current worldwide scenario, herbal remedies, products, and supplements have shown tremendously increasing demand and acceptance over the past four decades. Medicinal plants play vital roles in disease prevention; thus, their promotion with conscious efforts towards recognizing, identifying, and scrutinizing the ethnobotanical data for their medicinal uses [1]. Melghat region, best known as Melghat Tiger Reserved (MTR), has a unique ecological niche and conserved forest region in the Maharashtra state of India. Over the period, many forest officers, botanists, and researchers explored the Melghat regionand documented the Melghat Flora [2]. Pioneering work on identification and description varied floristic of the Central Provinces, and Berar was carried out by Witt (1916) and after that by Patel RI [3]. The first exhaustive work was published vide Technical Bulletin No 1, a document named "Flora of Melghat Tiger Reserve" [4], which described 647 naturalized species belonging to 398 genera of 97 families. A further addition to this biodiversity list was made vide Technical Bulletin No VII as "Additions to the Flora of Melghat"[5]. Their work continued for one and a half years, resulting in the addition of 67 species to the earlier reports of Angiosperms. Further, scientists from the Botanical Survey of India Western Region, Pune, studied the flora of Melghat and added 58 species [6]. Recently "Checklist of Flora of Melghat: 2018-19" enlisting 117 families, 547 genera, and 1008 species, more than 550 species were reported for ethnobotanical and pharmacological values [2]. Sensing the need and opportunity for review and digitize the available past floristic documents on Melghat Flora, this databank "Melghat Plant databank (MPdb)" was created to explore more possibilities on medicinal plants, phytochemicals, and further herbal drug research.

## Methodology:

## Description:

Pre-defined criteria [7] were considered for selecting the research articles. The published literature and information from international scientific databases such as PubMed, PubMed Central, Medline, Science Direct, Scopus, Google Scholar, Research Gate, and Web of Science were reviewed [8]. The plant's botanical name, synonyms, families, classification, and medicinal uses were verified using authorized books, published research articles, and publicly available online sources [9]. The Plant List web server [10] was utilized to identify the plant synonyms and reduce redundancies.

## Database design:

The Melghat Plant Databank (MPdb) has been developed in HTML, CSS, MySQL, and PHP. While the webpages were designed in HTML and CSS, the backend database was built in MySQL, and for the server-side pre-processing, PHP was used. AJAX scripting was utilized to improve auto-search and auto-suggest user search queries.

## MPdb features:

The MPdb is a user-friendly online free web database (Figure 1 - see PDF), wherein the user can search plant records by "Botanical Name", "Common Name", or "Family". Search results facilitate the user to select the entry of interest from displayed records, and the user can further explore the respective record. The total number of plants and medicinal plants were categorized (Table 1- see PDF) into eight categories per their habitat. Users can select their respective categories as well. Each MPdb record consists of the databank id, botanical name, synonyms, common names, category, family, classification, location, and current status, plant description, medicinal uses, reported phytochemicals, and published cross references. 

## MPdb dashboard:

The MPdb database also provides an interactive dashboard available at http://mpdb.co.in/dashboard/. It summarizes the data with criteria of the plant family, category, genus, medicinal, and non-medicinal record count information about the MPdb databank. This dashboard has been developed using Google Data Studio.

##  Caveat and future development:

With the above previews, our future objectives for MPdb are to review and update the phytochemical for complete Melghat Flora, with physiochemical information, structural information, druggability information, and bioactivities for possible phytochemicals. Further, we also have plans to include ethnobotanical information for possible diseasesand plant parts used by ethnic communities with valid cross-published references for the same.

## Conclusion:

Melghat Plant Databank (MPdb) is a user-friendly online web database that records 1028 plant species from 139 families and 537 genera; among them, 660 species have medicinal values used for more than 140 different diseases. MPdb records also provide information (Table 2 - see PDF), as per IUCN RED LIST [11] species for Melghat Flora. The MPdb finds utility to the students, researchers, and aboriginal communities for a quick review of available medicinal plantsand their uses, and it further provides enormous scope for better-applied prospects in herbal drug research and product development.
